# Foliar treatments with *Gaultheria procumbens* essential oil induce defense responses and resistance against a fungal pathogen in *Arabidopsis*

**DOI:** 10.3389/fpls.2014.00477

**Published:** 2014-09-23

**Authors:** Sophie Vergnes, Nathalie Ladouce, Sylvie Fournier, Hicham Ferhout, Faouzi Attia, Bernard Dumas

**Affiliations:** ^1^Université de Toulouse, UPS, Laboratoire de Recherche en Sciences VégétalesCastanet-Tolosan, France; ^2^CNRS, Laboratoire de Recherche en Sciences VégétalesCastanet-Tolosan, France; ^3^Natex BiotechToulouse, France; ^4^Equipe Recherches Agronomiques, AgronutritionCarbonne, France; ^5^LabCom C2R-BIONUTToulouse, France

**Keywords:** methyl salicylate, *Arabidopsis*, dynamic array, essential oils, plant immunity

## Abstract

Essential oil from *Gaultheria procumbens* is mainly composed of methylsalicylate (MeSA) (>96%), a compound which can be metabolized in plant tissues to salicylic acid, a phytohormone inducing plant immunity against microbial pathogens. The potential use of *G. procumbens* essential oil as a biocontrol agent was evaluated on the model plant *Arabidopsis thaliana*. Expression of a selection of defense genes was detected 1, 6, and 24 h after essential oil treatment (0.1 ml/L) using a high-throughput qPCR-based microfluidic technology. Control treatments included methyl jasmonate and a commercialized salicylic acid (SA) analog, benzo(1,2,3)-thiadiazole-7carbothiolic acid (BTH). Strong induction of defense markers known to be regulated by the SA pathway was observed after the treatment with *G. procumbens* essential oil. Treatment induced the accumulation of total SA in the wild-type *Arabidopsis* line Col-0 and analysis of the *Arabidopsis* line *sid2*, mutated in a SA biosynthetic gene, revealed that approximately 30% of MeSA sprayed on the leaves penetrated inside plant tissues and was demethylated by endogenous esterases. Induction of plant resistance by *G. procumbens* essential oil was tested following inoculation with a GFP-expressing strain of the *Arabidopsis* fungal pathogen *Colletotrichum higginsianum*. Fluorescence measurement of infected tissues revealed that treatments led to a strong reduction (60%) of pathogen development and that the efficacy of the *G. procumbens* essential oil was similar to the commercial product BION^®^. Together, these results show that the *G. procubens* essential oil is a natural source of MeSA which can be formulated to develop new biocontrol products.

## Introduction

Plant are constantly challenged by harmful microorganisms and diseases caused by fungal, bacterial, or viral phytopathogens pose severe threats to crop productivity worldwide (Fisher et al., 2012). Controlling plant diseases requires the use of massive amounts of synthetic pesticides, the breeding of resistant plant varieties, and agronomical strategies such as crop rotation. The development of an environmental-friendly and sustainable agriculture drives the search for alternative strategies and among these the use of natural compounds able to stimulate the plant immune system.

Plants possess the intrinsic capacity to respond efficiently to pathogen attacks by mounting immune responses. The first line of microbial perception occurs through the detection of molecular patterns exposed or released by microbial molecules and named PAMPs (or MAMPs; Pathogen (or Microbial) Associated Molecular Patterns). These molecular patterns interact with specialized receptors and the perception of PAMPs induced a signaling cascade which culminates with the expression of defense genes (Boller and Felix, 2009).

Thus, inducing resistance to pathogens by mimicking PAMPs activity could represent an attractive strategy to protect plants against diseases. Several products from natural sources, mainly algal polysaccharides, have been used as plant defense activators. These include β 1,3 glucan from the brown algae *Laminaria digitata*, ulvans from the green algae *Ulva* sp. sulfated fucans and carrageenans (Klarzynski et al., 2000, 2003; Mercier et al., 2001; Jaulneau et al., 2010, 2011). However, probably the most successful commercial compound inducing plant defense reactions is the chemical benzo(1,2,3)-thiadiazole-7carbothiolic acid (BTH) (also named acibenzolar-S-methyl, ASM) (Lawton et al., 1996). BTH has been shown to efficiently control diseases on several crops in field experiments demonstrating that eliciting plant defenses can constitute an alternative strategy to chemical fungicides (Walters et al., 2013). In fact BTH is a functional analog of the phytohormone salicylic acid (SA), a major signaling compound controlling the development of a part of immune responses in concert with jasmonic acid (JA) and ethylene. A tightly balance hormonal control determine the type of defense against a given pathogen (Bari and Jones, 2009). SA signaling pathway has been implicated in the induction of defense against biotrophic pathogens and acts antagonistically of the JA pathway involved against necrotrophs (Glazebrook, 2005). SA produced at the infection site is the initial signal which engages the systemic induction of defense responses, a phenomenon called Systemic Acquired Resistance (SAR). Construction of transgenic plants expressing a bacterial salicylate hydroxylase able to convert SA to the inactive product catechol, demonstrated the essential role of SA in SAR (Delaney et al., 1994). In *Arabidopsis*, accumulation of SA induces the nuclear localization of the central immune regulator Non Expressor of Pathogenesis-Related Protein 1 (NPR1). NPR1 is a transcriptional regulator which controls the expression of a wide range of SAR-related genes, among these genes coding the Pathogenesis-Related 1 protein (PR1) (Pajerowska-Mukhtar et al., 2013).

Two biosynthetic pathways are involved in the biosynthesis of SA derived from phenylalanine or chorismic acid. In *Arabidopsis*, the chorismic acid pathway is the major route to SA. Chorismic acid is the substrate of isochorismate synthases (ICSs) which produce isochorismate, a direct precursor of SA. The essential role of ICSs genes has been demonstrated by the isolation of mutants such as *sid2* (Wildermuth et al., 2001). In *sid2* mutants, accumulation of SA upon pathogen infection reaches only 5–10% of wild type level (Wildermuth et al., 2001). However, it has been shown from grafting experiments that SA is not the mobile signal inducing SAR (Vernooij et al., 1994). Several mobile signals have been identified including the methylated SA derivative, methyl SA (MeSA), a dicarboxylic acid, azelaic acid (AzA), an abietane diterpenoid, dihydroabetinal (DA), and a phosphorylated sugar derivative, glycerol-3-phosphate (G3P) (Shah et al., 2014). While the function of these metabolites depends on SA, only MeSA is directly linked to SA synthesis since this compound is produced upon the transfer of a methyl group from the donor S-adenosyl methionine methylation catalyzed by a SA-methyltransferase (SAMT). In tobacco, silencing of a SAMT gene and grafting experiments demonstrated the essential role of MeSA synthesis in the development of SAR upon infection with the tobacco mosaic virus (Park et al., 2007). In the distal leaves, MeSA is converted to SA by the action of MeSA esterases, enzymes which has been shown essential for the expression of SAR in tobacco leaves (Aviv et al., 2002; Park et al., 2007).

Since SAR can protect efficiently plants against a wide range of microbial pathogens, molecules which induce this response can represent an attractive strategy to protect plants. The main advantage of this strategy is the use of non-toxic natural products instead of chemical fungicides which are harmful for the environment. However, the induction of SAR through the salicylate pathway for use in organic agriculture would require the identification of natural sources of active compounds. The plant genus *Gaultheria* (*Ericaceae*) includes several species largely present in Asia and North America, growing as evergreen shrub. Most *Gaultheria* species are regarded as traditional herbal medicine, notably used in the treatment of pain. Highly relevant to this biological activity is the composition of *Gaultheria* essential oil (GEO) which contains more than 96% methylsalicylate (MeSA) and several derivatives of MeSA has been identified (Liu et al., 2013; Nikolic et al., 2013).

Here, we used a new high-throughput Q-PCR microfluidic method to analyze in a single Q-PCR run the expression of biomarker genes related to plant defense after treatment of *Arabidopsis* plant with GEO. Determination of SA levels after treatment of *sid2* plants showed that MeSA from GEO is readily absorbed and metabolized by plant tissues. Our results demonstrate that GEO is a natural source of MeSA which can substitute to synthetic SA analogs for agronomic applications.

## Materials and methods

### Plant, fungal strains, and chemicals

*Arabidopsis thaliana* lines used were Col-0 wild-type, transgenic line PR1-GUS (Shapiro and Zhang, 2001), *sid2* (Wildermuth et al., 2001), and *NahG* mutant plants. For treatment assays, seeds were soaked in water for 40 min, and then treated for 30 min in 2.4% sodium hypochlorite solution, followed by four washings in sterile water. The surface-sterilized seeds were transferred into wells of 24-well microtiter plates (2 seeds per well), containing 300 μl of Murashige and Skoog medium (MS; Sigma-Aldrich) supplemented with 1% sucrose. The plates were incubated under 16 h illumination period (270 μmol m^−2^ s^−1^) and 8 h night period at 23°C on a rotary shaker at 90 rpm for 3 days and then on a static tray for 12 days. For SA analysis and pathogenicity tests, plants were grown on pots (Jiffy, Lyon, France) in a growth chamber under 12 h light (270 μmol m^−2^ s^−1^), at 23°C and 12 h dark at 20°C during 3 weeks.

A *Colletotrichum higginsianum* transgenic strain expressing the green fluorescent protein (O'Connell et al., 2004) was used to pathogenicity assays. The strain was grown on Mathur's agar medium (glucose 2.8 g/L; MgSO_4_ 1.22 g/L; KH_2_PO_4_ 2.72 g/L; peptone 2.8 g/L; Agar 30 g/L) in the dark at 23°C. After 10 days of growth, fungal spores were recovered with sterile water and the concentration was adjusted after counting the spores. GEO and synthetic methyl salicylate were obtained from Nat'Ex Biotech (Toulouse, France). Methyl jasmonate was obtained from Sigma-Aldrich. Flg22 peptide was synthesized by Proteogenix (Strasbourg, France) with purity greater than 90%. Chitin oligomers obtained from Crab shell chitin (CSC, Sigma-Aldrich) were prepared as described by Nars et al. (2013). BTH formulated as BION® was obtained from Syngenta.

### Plant treatments

For the treatment of plant grown in 24-well microtiter plates, chemicals were added in the liquid MS medium after 10 days of growth. Foliar treatments were done on 3-week old plants grown on pot by spraying 1 ml/pot of the chemicals using a manual spray.

### GUS activity assays

Total proteins were extracted from 100 mg of treated plants after grinding in liquid nitrogen, with 100 μl of GUS buffer (100 mM Phosphate buffer pH7, 0.1% TritonX-100, 10 mM β-mercaptoethanol) (Jefferson et al., 1987). Glucuronidase activity was measured by fluorometric assay with 25 μl of protein extracts and 1 mM MUG (4-methylumbelliferyl glucuronide, Sigma) in a total reaction volume of 200 μl. Fluorescence was measured every 5 min during 120 min on a TriStar LB 941 Multimode Microplate Reader (Berthold Technologies) at 37°C with 360 nm excitation and 460 nm emission. The fluorimeter was calibrated with freshly prepared MU (4-methylumbelliferone sodium salt, Sigma-Aldrich) standards in the same GUS buffer. Protein concentration was determined by the method of Bradford on 96 well plates. Two hundred microliter of Bradford reagent (Bio-Rad Laboratories) were added to 10 μl of samples. After incubation (15 min, 25°C), absorbance was measured at 565 nm. Standard curve was done with 1–20 μg of BSA (Sigma-Aldrich). Glucuronidase activity was calculated from the linear part of the reaction (between 20 and 100 min) and expressed as nkatal/mg of total proteins.

### Gene expression analysis

Total RNAs were extracted using the SV Total RNA Isolation System kit (Promega®, Charbonnières, France). For each sample, 1 μg of total RNA was reverse-transcripted with the High-Capacity cDNA Reverse Transcription Kit (Applied Biosystems®, Courtaboeuf, France). High-throughput Q-PCR was performed using the BioMarkTM HD System (Fluidigm®, Issy les Moulineaux, France). Briefly, cDNAs were diluted to ~50 ng/μl prior to be submitted to specific target amplification (STA) by 14 cycles of PCR amplification (95°C for 15 s and 60°C for 4 min) in a reaction mix containing the 96 primer pairs (50 nM) and the TaqMan® PreAmp Master Mix (1:2) (Applied Biosystems®, Courtaboeuf, France). Primer sequences are presented in the Table S1. Pre-amplified cDNAs were diluted with TE buffer (1:5) and used for Q-PCR array analysis in a reaction mix containing TaqMan Gene expression Master mix, DNA Binding Dye Sample Loading Reagent and EvaGreen®. Data were analyzed with the BioMark Real-Time PCR Analysis Software Version 2.0 (Fluidigm). The NormFinder software (Andersen et al., 2004) was used to determine the best housekeeping genes. Relative gene expression was calculated over three independent experiments and significant gene deregulations were determined by student *t*-test.

### SA analysis

For determination of SA content, 100 mg of leaf tissue were ground in liquid nitrogen, and 50 ng of an internal standard (o-anisic acid, oANI) were added before the extraction of total SA. Total SA (free SA and SA conjugates) was extracted by methanolic extraction followed by acid hydrolysis. The hydrolysate was then subjected to organic phase partitioning (ether). After extraction, organic phase was evaporated and sample diluted with 100 μl of acetonitrile/water/formic acid (50/50/0.1%, v/v/v). Analysis was performed with a reversed phase high-performance liquid chromatography (HPLC, Ultimate 3000, ThermoScientific) coupled with fluorescence detection (Jasco FP-920). SA and oANI were separate on a XBridge reverse phase column (25 cm × 4.6 mms × 5 μm, Waters) and a XBridge guard column (2 cm × 4.6 mm × 5 μm, Waters) by gradient elution with a binary system of acetonitrile-water-formic acid. Mobile phase consisted of acetonitrile-formic acid (100: 0.1%, v/v) and water-formic acid (100: 0.1%, v/v) at a flow rate of 0.8 mL/min. Fluorescence detection set at excitation/emission wavelengths 294/359 nm for oANI and 305/407 nm for SA. SA was quantified using Chromeleon 6.8 chromatography software (ThermoScientific). Corrections for losses were made for each individual sample according to recoveries of the internal standard.

### Pathogenicity tests

Infection tests were performed on 3 weeks old *A. thaliana* Col-0 plants. Foliar tissues were sprayed with BION® or GEO and inoculation was done 48 h after treatment. A spore suspension of *C. higginsianum-GFP* at 10^5^ spores/ml was sprayed on the plants. The fungal colonization was evaluated after 7 days by GFP quantification. Proteins were extracted from 100 mg of five aerial parts of plants after grinding in liquid nitrogen, with 200 μl of extraction buffer (100 mM Phosphate buffer pH7, 0.1% TritonX-100, 100 mM NaCl, 10 mM EDTA, and 1 mM PMSF). GFP fluorescence was measured on 200 μl of protein extracts with 485 nm excitation and 535 nm emission during 1 min on TriStar LB 941 Multimode Microplate Reader (Berthold Technologies) and expressed in RFU (Relative Fluorescence Unit).

## Results

### Dose-dependent induction of the SAR marker PR1 by GEO

**Figure 1:**
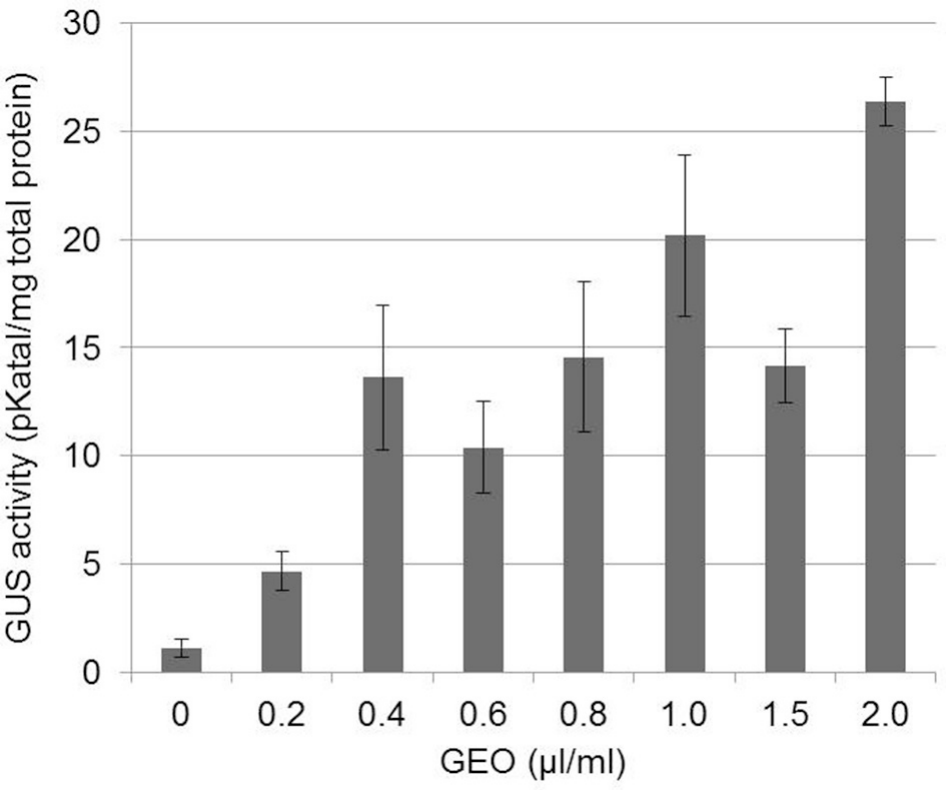
**Dose-dependent induction of PR1::GUS gene by GEO foliar treatments.** PR1::GUS *Arabidopsis* leaves were treated with various concentrations of GEO. GUS activity was determined 48 h after the treatment. Three independent biological experiments were performed for each condition. Bars: standard error to the mean.

As a first approach to study GEO elicitor activity, PRI-GUS plants (Shapiro and Zhang, 2001) grown in microtiter plates were incubated in GEO solutions at various concentrations and β-glucuronidase activity was measured 48 h after treatment. A 5 fold increase in β glucuronidase activity was observed at the lowest GEO concentration (0.2 μl/ml), reaching a maximum activity (25 fold increase) at 2 μl/ml with no symptom of phytotoxicity (Figure 1). A concentration of 1 ml/L was chosen for further studies.

### Induction of gene expression upon GEO treatments

**Figure 2:**
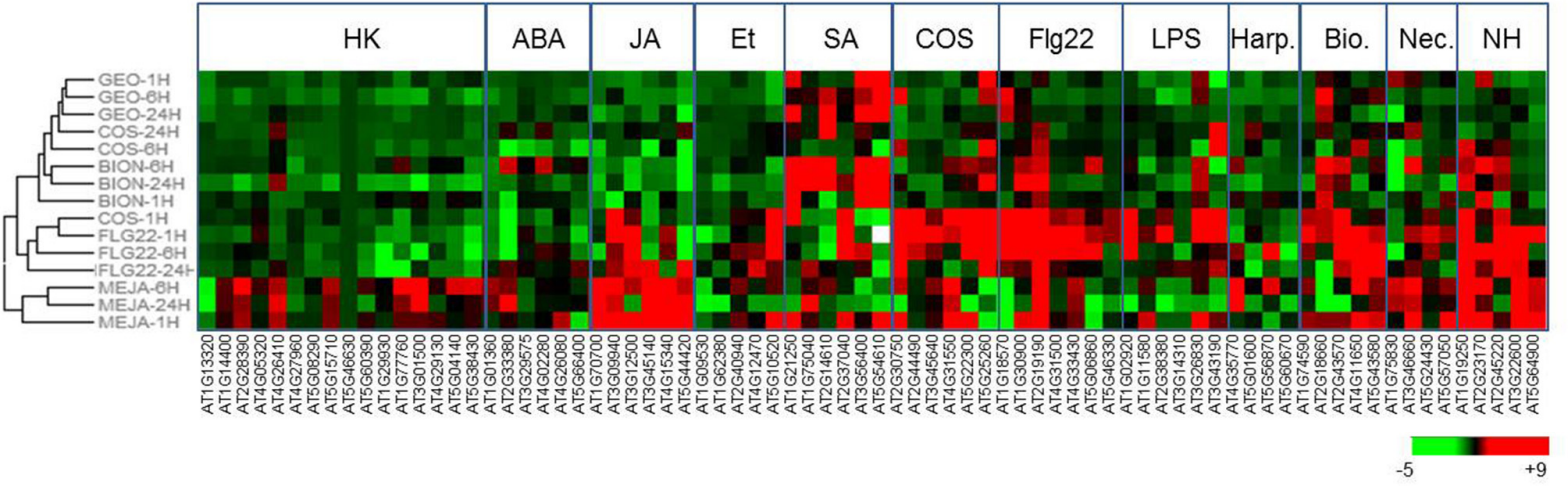
**GEO treatment induces SA-regulated defense genes.** Gene expression after GEO treatment of *Arabidopsis* leaves with various compounds (MeJA, SA, FLG22, COS, BION, GEO) was analyzed using the microfluidic dynamic array. Treatments were clustered using the HCE clustering software and default parameters.

To construct a diagnostic chip to monitor plant defense responses, marker genes corresponding to various classes of immune responses were selected by mining transcriptomic databases (Winter et al., 2007; Hruz et al., 2008) and bibliographic data (Table S2). The categories selected included genes induced by phytohormones (SA, JA, ethylene, and absicic acid), by microbial PAMPs (FLG22, chitosaccharides, harpin, and lipopolysaccharides), pathogen infection and housekeeping genes. To validate the selection of marker genes, *Arabidopsis* plants (Col-0) grown in microtiter plates were treated with solutions containing mehyljasmonate (MeJA), FLG22 peptide, BTH (BION® formulation), and chitosaccharidic fragments obtain from crab shell chitin (DP 6-9) (Nars et al., 2013). RNAs were extracted 1, 6, and 24 h after treatments and gene expression was analyzed by real-time quantitative-RT PCR using the dynamic array (Figure 2). Normalization was done using the standard gene AT5G46630 encoding a clathrin adaptor complex medium subunit family protein, selected upon analysis of the results with the Normfinder algorithm (Andersen et al., 2004). Genes falling in the JA and SA category were mainly up-regulated following the corresponding treatment (MeJA and BION®, respectively), excepted for the WAK1 gene (At1G21250) and the ankyrin gene (At5G54610) which showed a strong up-regulation 1 h after treatment by both phytohormones. Similar induction of PAMP-induced markers was obtained following Flg22 and COS treatment. Together these results show that the selection of marker genes used combined with the microfluidic dynamic array is a useful approach to determine the activity of plant immunity elicitors.

**Figure 3:**
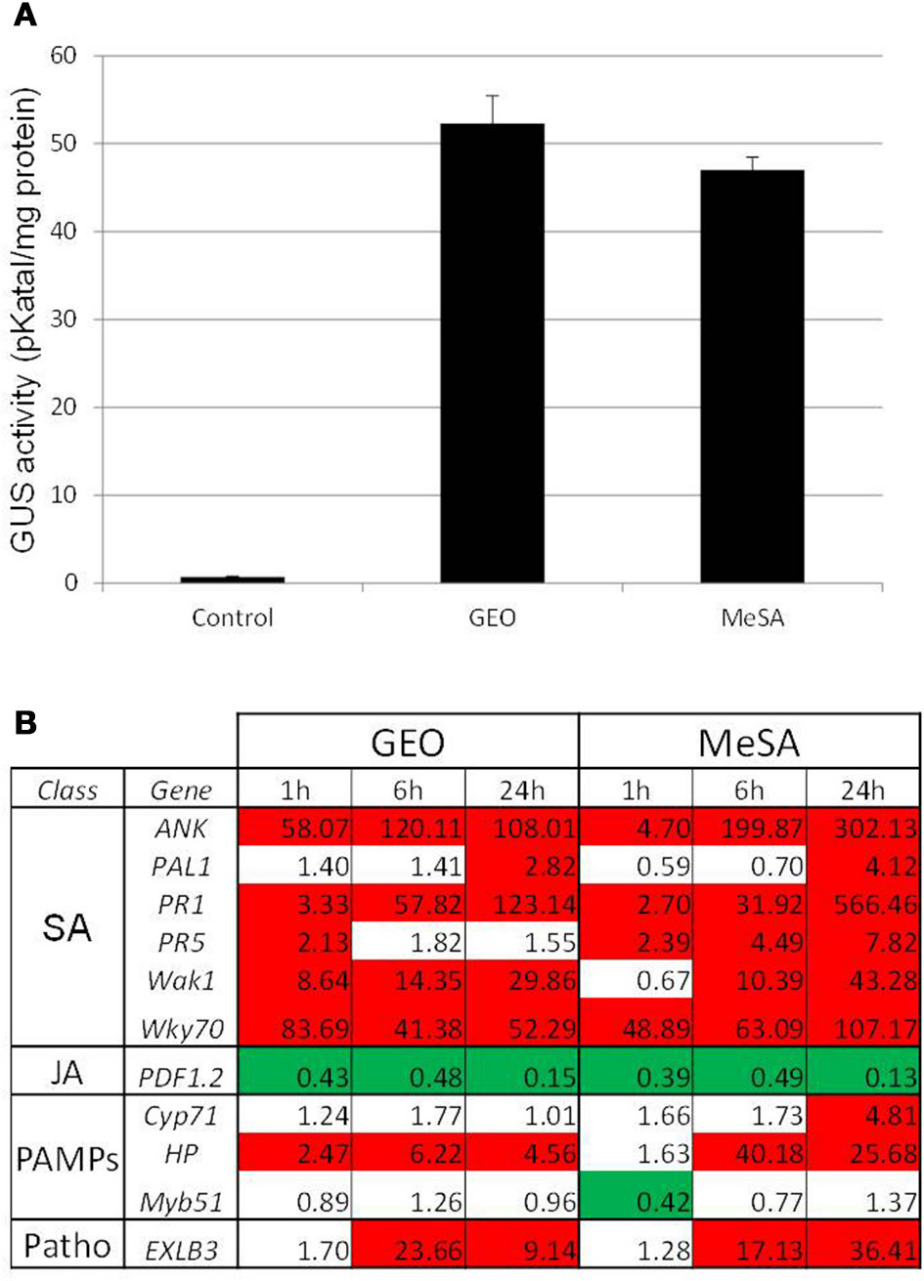
**GEO and MeSA show similar activity on defense gene expression.** PR1::GUS *Arabidopsis* leaves were treated with GEO or MeSA solution at 1 μl/ml. GUS activity **(A)** was analyzed 48 h after treatments and defense gene expression **(B)** was analyzed by Q-PCR 1, 6, and 24 h post-treatments. Control plants were treated with a solution containing the wetting agent.

The dynamic array was used to determine the activity of GEO. The product was mixed with a wetting agent and spray at 0.1% final concentration on Col-0 leaves. Expression of marker genes by the microfluidic dynamic array was determined 1, 6, and 24 h after the treatment. GEO strongly induced SA marker genes but not genes falling into other categories (Figure 2) suggesting that GEO activity is mainly due to MeSA. To confirm this result, GEO activity was compared with the activity of pure MeSA. β-glucuronidase activity from GEO or MeSA treated-PR1::GUS plants were analyzed 48 h after the treatment. A similar induction of the glucuronidase activity was observed (Figure 3). Expression of a selection of defense genes was analyzed by real-time quantitative RT-PCR. Again, similar values were obtained in both treatments showing that GEO display a comparable activity to MeSA (Figure 3).

### Accumulation of salicylic acid in wild-type and *sid2* plants treated with GEO

**Figure 4:**
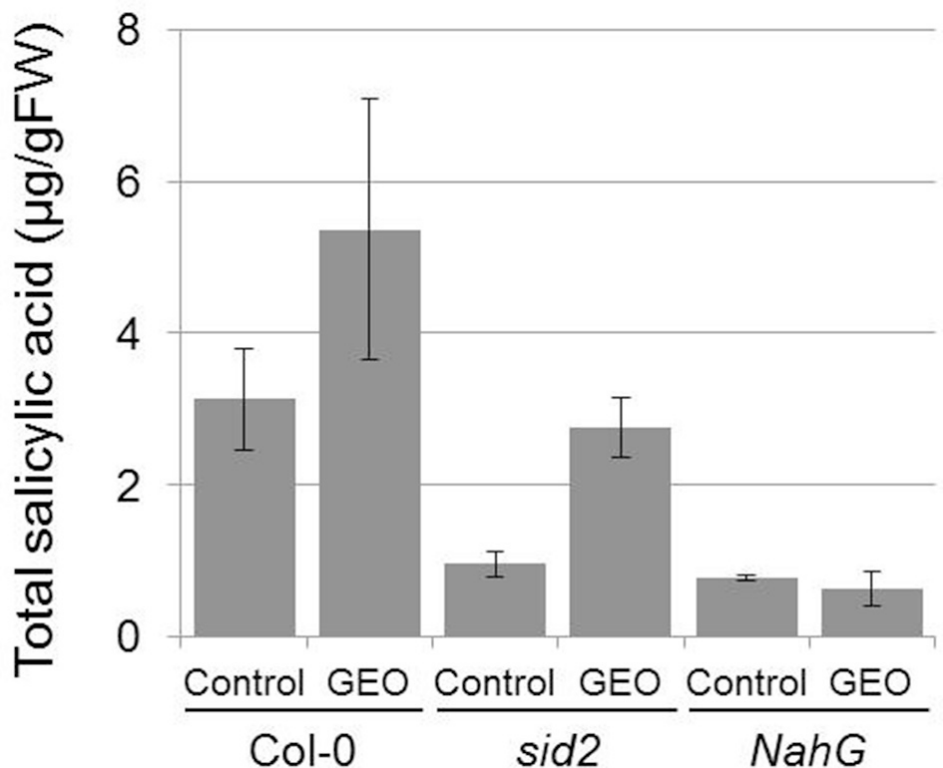
**GEO treatments increase total salicylic acid content.** Col-0, *sid2*, and NahG plants were treated with GEO at 1 μl/ml. Total SA was determined 4 days after treatments. Two biological replicates were analyzed with similar results. Bars: standard errors to the mean.

The effect of GEO on SA accumulation was studied on wild-type Col-0 plants, *sid2* plants mutated in the gene coding the SA biosynthetic enzyme ICS (Wildermuth et al., 2001) and *NahG* plant expressing a bacterial SA hydroxylase. The rationale behind the use of *sid2* plants was to determine the proportion of external MeJA which accumulate inside the plant tissues and subsequently demethylated by endogenous esterases. Treatments of Col-0 plants with GEO solution led to a strong increase of total SA content in treated leaves. The background level of SA was found to be lower in control *sid2* and *NahG* plants compared to Col-0. However, treatment with GEO led to an increase of SA content only in *sid2* plants and not in *NahG* leaves (Figure 4). This suggests that MeSA from GEO penetrated *sid2* leaves and was efficiently demethylated by endogenous SA esterases.

### Treatment with GEO protects plant to fungal infection

**Figure 5:**
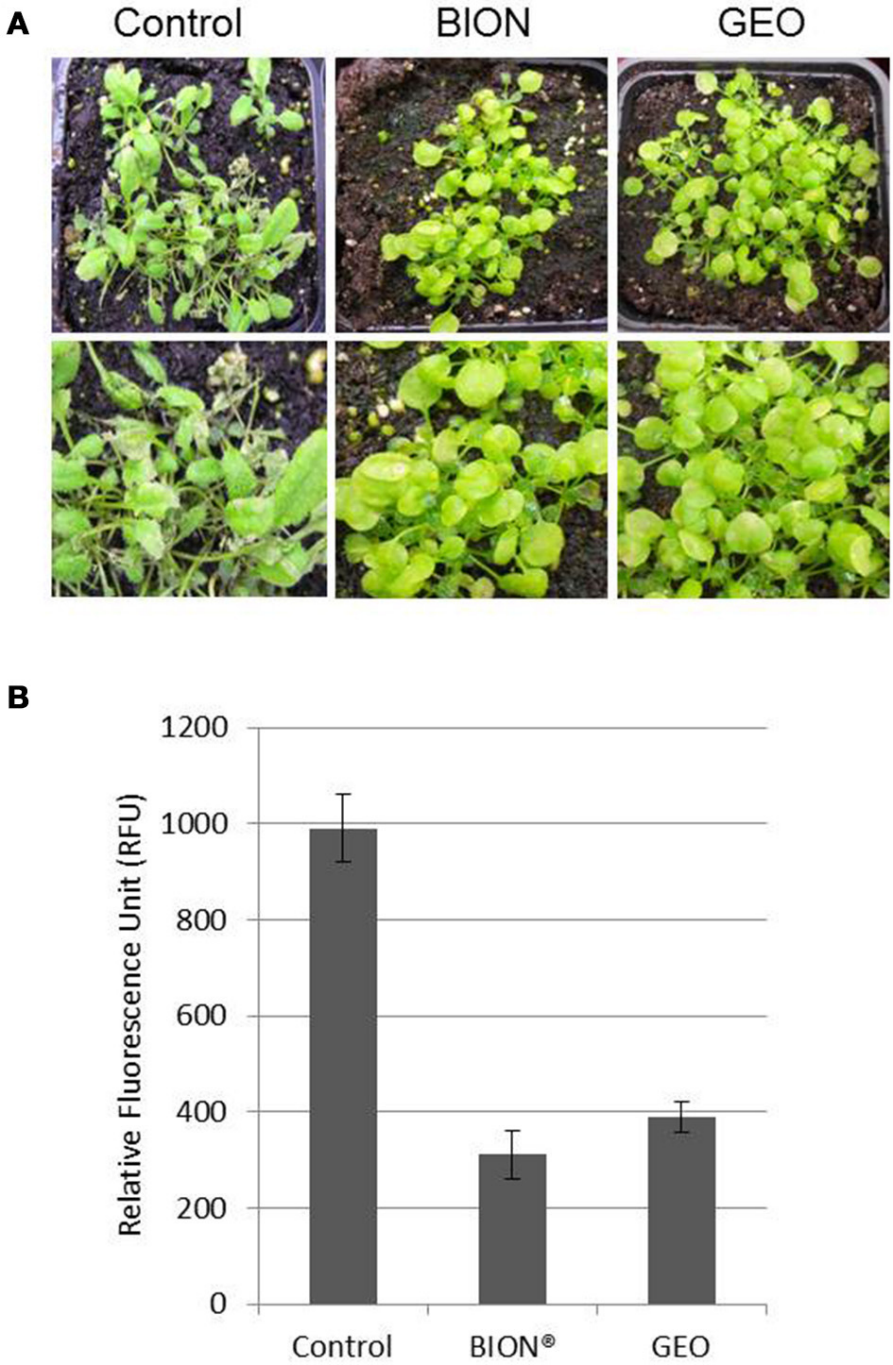
**GEO treatment induces resistance against the foliar pathogen *C. higginsianum*.** Col-0 plants were treated with GEO at 1 μl/ml or BION (40 μg/ml) and inoculated with a conidial suspension of a GFP-expressing strain of *C. higginsianum* 48 h later. Macroscopic symptoms **(A)** and GFP fluorescence **(B)** were analyzed 7 days after inoculation.

**Figure 6:**
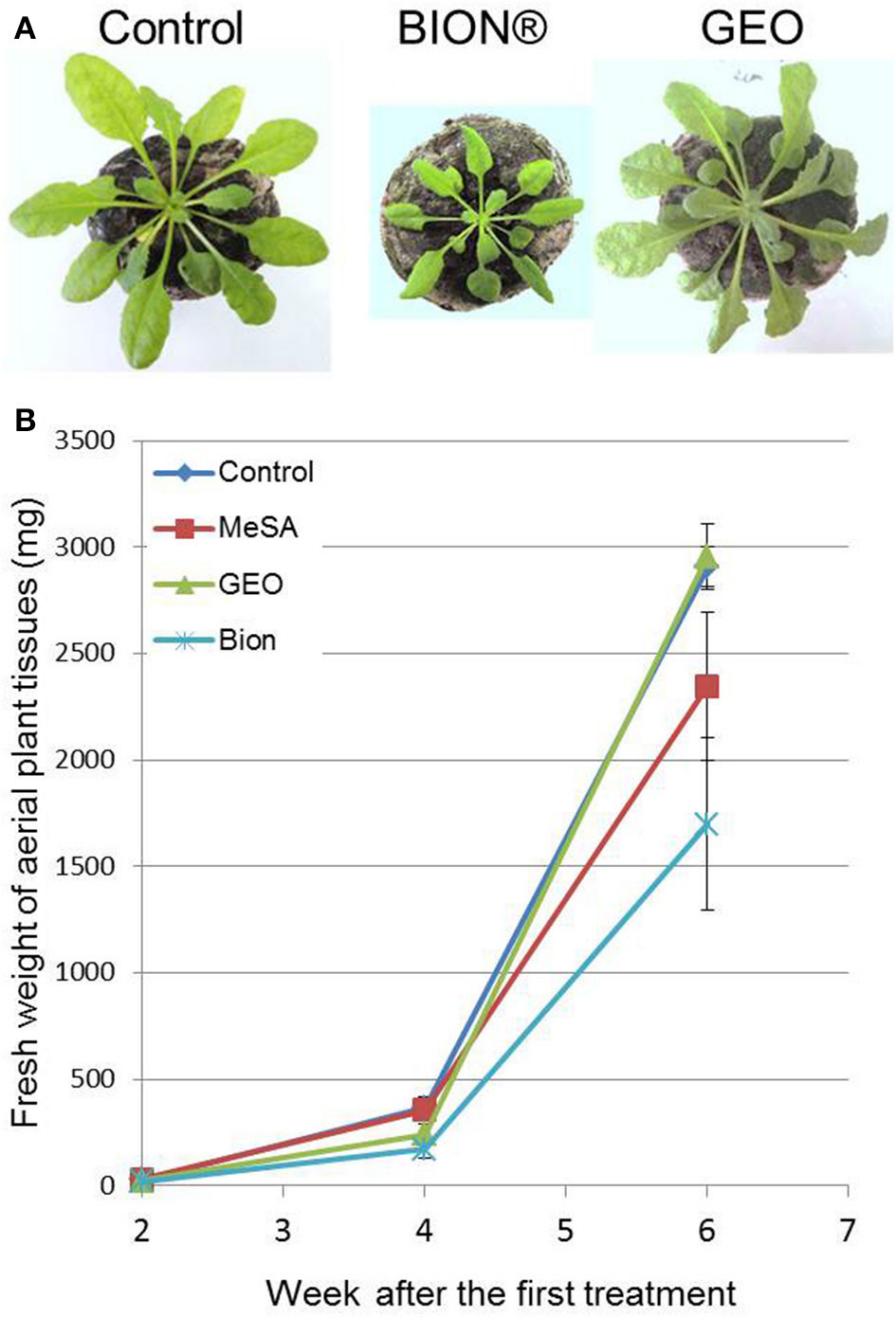
**Effect of successive GEO treatments on plant development.** Fifteen-days old plants were treated weekly with wetting agent, Bion^®^ (40 μl/ml), and GEO (1 μl/ml) during 6 weeks. Photos **(A)** were taken after 6 weeks and fresh weight of plant tissues **(B)** was determined at 2, 4, and 6 weeks after the first treatment. Bars: Standard error to the mean.

To evaluate the effect of GEO treatment on fungal disease, we used the *Arabidopsis* pathogen *C. higginsianum* (O'Connell et al., 2004). Col-0 plants treated with the wetting agent or with a solution containing GEO at 0.1% were inoculated with a conidial suspension of a GFP-expressing *C. higginsianum* strain. Six days after inoculation, BION® and GEO treated-leaves showed reduced symptoms compared to control plants (Figure 5). Quantification of GFP-fluorescence indicated a reduction of about 60% of the fungal development in treated-leaves (Figure 5). Additionally, the effect of successive GEO, MeSA, and BION® treatments on plant development was also evaluated over a 6 weeks period. It was previously shown that successive treatment of *Arabidopsis* with BION® can affect several fitness parameters (Van Hulten et al., 2006). Weekly treatments of GEO and MeSA did not induce a detectable effect on plant development (Figure 6). However, weekly BION® treatments led to decrease of about 50% of aerial part weight of the plants in agreement with previous results (Van Hulten et al., 2006).

## Discussion

Here, we investigate the biological activity of an essential oil from the medicinal plant *Gaultheria procumbens*. Expression analysis showed that treatment of *Arabidopsis* leaves induced SA-dependent genes, similarly to the SA analog BTH, the active ingredient of the commercialized product BION®, and to MeSA. The potential use of essential oils for the control of plant diseases has been mainly linked to a direct effect on the growth of pathogenic fungi (Yoon et al., 2013). However, a still largely unexplored effect of essential oils could be their activity on plant immunity. While a direct effect of high concentration of GEO on the growth of filamentous fungi was recently reported (Nikolic et al., 2013), we did not observe an inhibitory effect of *C. higginsianum* growth at a concentration of 1 μl/ml (not shown). These results suggest that MeSA is the main active ingredient of GEO through the induction of SA-dependent immune responses resulting in an increase resistance to pathogenic fungi.

The development of a collection of marker genes representing various classes of immune responses as well as housekeeping genes and the use of a microfluidic high-throughput real-time reverse transcription-Q-PCR platform [the BioMark HD (TM) system from Fluidigm] allowed us to identify the mode of action of GEO. This technology enabled the analysis in a single Q-PCR run of the expression of 96 markers in 96 cDNA preparations. To our knowledge, this system was never used to study the activity of compounds active on plants. While our selection of marker genes was dedicated to identify compounds inducing immune responses, this approach could be used to characterize other types of compounds acting on resistance against abiotic stress or stimulating developmental processes. Thus, the Biomark system could represent an attractive and cost-effective alternative method to other systems based on the use of microarrays (Von Rad et al., 2005).

To determine whether GEO treatment allowed the accumulation of SA inside plant tissues, we used the *sid2* mutant defective in the ICS involved in the main SA biosynthetic pathway in *Arabidopsis* (Wildermuth et al., 2001). Treatment of *sid2* leaves led to accumulation of SA suggesting that MeSA from GEO is efficiently demethylated by *A. thalian*a MeSA esterases. Multiple esterases able to efficiently demethylate MeSA have been identified in *A. thaliana* (Vlot et al., 2008). The effect of GEO on induced resistance was measured on *Arabidopsis* leaves inoculated with the fungal pathogen *C. higginsianum*. It has been previously shown that *Arabidopsis* plants expressing the bacterial salicylate hydroxylase gene *NahG* are hypersusceptible to *C. higginsianum* suggesting a role of the SA-dependent pathway in the defense against this fungus (O'Connell et al., 2004). Additionally, benzothiadazole treatments have been shown to reduce anthracnose symptoms on various plants further supporting a role of the SA pathway in *Colletotrichum* immune responses (Bigirimana and Hofte, 2002; Smith-Becker et al., 2003; Zhu et al., 2008). Thus, this pathosystem was very well-suited to evaluate the impact of GEO treatments on SA-induced plant resistance. In our experimental conditions, GEO was as effective as BION® to reduce *C. higginsianum* development.

Interestingly, GEO treatments did not strongly modify the expression of housekeeping genes whereas significant repression of genes coding enzymes involved in primary metabolism was observed after treatment with BION®. Correlatively, no effect on plant growth was observed after repeated application of GEO whereas a significant reduction of plant biomass was obtained with BION® treatments. The effect of benzothiadazole treatments on plant development is well-documented and has been associated to a reduction of plant growth and seed production in several plant species (Heil et al., 2000; Cota-Arriola et al., 2013). Negative effects of SA or its synthetic analogs has been attributed to toxic effects of SA and allocation costs (Vos et al., 2013). It is thus surprising that negative effects on plant development were not observed upon GEO treatments. While this point deserves further experimental work, it can be hypothesized that GEO treatment leads to a progressive accumulation of SA inside plant tissues since it required a demethylation step to be converted into an active product. This is not the case for BTH which is directly active on SA receptors (Fu and Dong, 2013).

To conclude, we show here that the essential oil from *G. procumbens* could be a valuable natural source of MeSA for biocontrol applications. MeSA has been detected in the composition of essential oils from various sources (Flamini et al., 2002; Nebie et al., 2004; Paudel et al., 2013; Radoias and Bosilcov, 2013) but in lower proportion than in GEO and associated with other metabolites which can display toxic effects from the plants or environmental organisms. However, additional assays are required to evaluate the activity of GEO in the field since activity of natural compounds can be challenged by environmental conditions and responses to abiotic stresses (Walters and Heil, 2007). Further studies will focus on the development of GEO formulations able to preserve GEO activity during product storage and field treatments.

## Author contributions

Sophie Vergnes and Bernard Dumas designed and performed the experiments and constructed the final manuscript. Sophie Vergnes and Nathalie Ladouce designed and performed the microfluidic Q-PCR experiment. Sophie Vergnes and Sylvie Fournier performed SA analyses and Hicham Ferhout prepared reagents. Bernard Dumas, Hicham Ferhout, and Faouzi Attia initiated and supervised the project.

### Conflict of interest statement

The authors declare that the research was conducted in the absence of any commercial or financial relationships that could be construed as a potential conflict of interest.
